# Childhood-onset systemic lupus erythematosus (cSLE) and malignancy: a nationwide multicentre series review

**DOI:** 10.1186/s42358-024-00353-3

**Published:** 2024-02-06

**Authors:** Matheus Zanata Brufatto, Sean Hideo Shirata Lanças, Taciana de Albuquerque Pedrosa Fernandes, Adriana Maluf Elias Sallum, Lucia Maria Arruda Campos, Ana Paula Sakamoto, Maria Teresa Terreri, Flavio Roberto Sztajnbok, Blanca Elena Rios Gomes Bica, Virginia Paes Leme Ferriani, Luciana Martins de Carvalho, Clovis Artur Almeida Silva, Claudia Saad-Magalhaes

**Affiliations:** 1 Department of Internal Medicine, Discipline of Rheumatology, Botucatu Medical School, São Paulo State University (UNESP), Botucatu, São Paulo Brazil; 2 Department of Pediatrics, Botucatu Medical School, São Paulo State University (UNESP), Botucatu, São Paulo Brazil; 3 Pediatric Rheumatology Unit, Child and Adolescent Institute, Hospital das Clínicas da Faculdade de Medicina da Universidade de São Paulo, São Paulo, Brazil; 4 Pediatric Rheumatology Unit, Universidade Federal de São Paulo, São Paulo, Brazil; 5 Pediatric Rheumatology Unit, Universidade Federal do Rio de Janeiro, Rio de Janeiro, Brazil; 6 Pediatric Rheumatology Unit, Universidade Federal do Rio de Janeiro, Hospital Universitário Clementino Fraga Filho, Rio de Janeiro, Brazil; 7 Department of Pediatrics, Ribeirão Preto Medical School, University of São Paulo, Ribeirão Preto, São Paulo Brazil; 8 Adolescent and Pediatric Rheumatology Units, Child and Adolescent Institute HC-FMUSP, Faculdade de Medicina da Universidade de São Paulo (FMUSP), São Paulo, Brazil; 9 Pediatric Rheumatology Unit, Department of Pathology, Botucatu Medical School, Sao Paulo State University (UNESP), Botucatu, São Paulo Brazil

**Keywords:** Childhood-onset systemic lupus erithematosus, Registry, Malignancy, Leukaemia, Lymphoma, Solid malignancy

## Abstract

**Background:**

Increased malignancy frequency is well documented in adult-systemic lupus erythematosus (SLE), but with limited reports in childhood-onset SLE (cSLE) series. We explored the frequency of malignancy associated with cSLE, describing clinical and demographic characteristics, disease activity and cumulative damage, by the time of malignancy diagnosis.

**Method:**

A retrospective case-notes review, in a nationwide cohort from 27 Pediatric Rheumatology centres, with descriptive biopsy-proven malignancy, disease activity/damage accrual, and immunosuppressive treatment were compiled in each participating centre, using a standard protocol.

**Results:**

Of the 1757 cSLE cases in the updated cohort, 12 (0.7%) developed malignancy with median time 10 years after cSLE diagnosis. There were 91% females, median age at cSLE diagnosis 12 years, median age at malignancy diagnosis 23 years. Of all diagnosed malignancies, 11 were single-site, and a single case with concomitant multiple sites; four had haematological (0.22%) and 8 solid malignancy (0.45%). Median (min–max) SLEDAI-2 K scores were 9 (0–38), median (min–max) SLICC/ACR-DI (SDI) score were 1 (1–5) Histopathology defined 1 Hodgkin's lymphoma, 2 non-Hodgkin's lymphoma, 1 acute lymphoblastic leukaemia; 4 gastrointestinal carcinoma, 1 squamous cell carcinoma of the tongue and 1 anal carcinoma; 1 had sigmoid adenocarcinoma and 1 stomach carcinoid; 3 had genital malignancy, being 1 vulvae, 1 cervix and 1 vulvae and cervix carcinomas; 1 had central nervous system oligodendroglioma; and 1 testicle germ cell teratoma.

**Conclusion:**

Estimated malignancy frequency of 0.7% was reported during cSLE follow up in a multicentric series. Median disease activity and cumulative damage scores, by the time of malignancy diagnoses, were high; considering that reported in adult series.

## Background

Childhood onset systemic lupus erythematosus (cSLE) occurs in a proportion of 10–15% of all SLE cases [1, 2]. It has been considered a more severe disease course, needing prolonged immunosuppressive treatment. Chronic and persistent inflammation may lead to genetic toxicity predisposing to oncogenesis [3]. Theoretically, the SLE autoantibodies can penetrate cells reaching nuclear genetic material, interfering with cell repair that could generate malignancy [4]. Besides the disease process itself, immunosuppressive treatment with cytotoxic drugs, could also predispose to malignancy [5].

Malignancy is considered damage, scoring at least 1point in the Systemic Lupus International Collaborating Clinics (SLICC)/American College of Rheumatology (ACR) Damage Index (SDI), with one point attributed to each of the malignancy sites [6, 7]. There are several reports of malignancy frequency in adult population, in several cohorts coming from different geographic region and environment. However, the reports about pediatric population, are very limited, restricted to only a few multicentric studies [8–15]. The estimates in a recent metanalysis showed that, cumulative frequency in cSLE was 0.5% before 18 years of onset age compared to 4.2% after 18 years of onset age [16]. Pediatric malignancies represent 2 to 3% of overall malignancy records in national registries [23].

Given the opportunity of examining a historical cohort for description of cSLE rare manifestations [17–20] we reviewed the database, searching for the frequency and specific diagnoses of malignancy, occurring after the cSLE onset.

## Method

A retrospective analysis of reported malignancy in patients with established cSLE diagnosis and follow up is presented. Cases were diagnosed before the age of 18 years, according to the American College of Rheumatology—ACR criteria (1997), by the attending physician in 27 pediatric rheumatology centres, convened by an established national study, the Brazilian cSLE Study Group Registry.

The data shared with participants included clinical descriptors of common and rare cSLE manifestations, demographics, clinical and standard laboratorial parameters, activity score by SLE Disease Activity Index 2000 (SLEDAI-2K) and damage scores by Systemic Lupus International Collaborating Clinics/ACR Damage Index (SLICC/ACR-DI) [6, 7], both scored by the attending physician in each of the centres, by the time of malignancy diagnoses. Damage accrual by SLICC/ACR-DI was scored only for those with more than 6 months follow up.

Data collection was established at diagnosis, one follow up visit and the last clinical visit, by the time of biopsy-proven malignancy diagnoses. The initial and the last visits were standardised, but the follow up visit occurred at variable time according to the physician assessor, for completeness of required data. The first two visits occurred before the malignancy diagnosis and the last follow up visit just by the time of diagnosis. Cases were retrospectively reviewed by investigators in each of the centres cohorts; data collection started in 2013 and finished in 2021.

Descriptive non-parametric statistics (median, minimum, maximum values) were used for reporting quantitative clinical and laboratorial variables. Categorical variables were described by frequency and percentages.

## Results

Of the 1757 patients from the c-SLE updated database, 12 (0.7%) reported malignancy. Of those, 11 were females, all with malignancy diagnosed during c-SLE follow up. According to the malignancy site involvement, 11 had a single site and one with more than one concomitant site. Median (min–max) age at cSLE diagnosis was 12(10–17) years; median (min–max) cSLE duration up to malignancy diagnosis was 10 (0.2–19) years and the mean (min–max) age at malignancy diagnosis was 23 (12–32).

Lymphoma and leukaemia were diagnosed in 4 cases; namely 1 with Hodgkin and 2 non-Hodgkin lymphomas; 1 acute lymphoblastic leukaemia. Sjögren’s syndrome association was not described in any of the patients in the malignancy series. Gastrointestinal tract malignancy was diagnosed in 4 cases, being squamous carcinoma of the tongue (1), anal carcinoma (1), sigmoid colon adenocarcinoma (1) and stomach carcinoid (1). Urogenital malignancy was seen in four, being vulva carcinoma (1), cervix carcinoma (1), vulva plus cervix carcinoma (1) and a germ line cell testicle teratoma (1). Central nervous system oligodendroglioma was reviewed in a single case.

The main signs and symptoms raising suspicious of malignancy were fever and pallor, described in hematopoietic malignancy cases; diarrhoea was observed in 2 of the cases with gastrointestinal malignancy; vulvar condylomas were observed in two cases during routine gynaecological exam; and a palpable testicle lump prompted the biopsy for diagnosing testicle teratoma (Table 1).

**Table 1:** Descriptive malignancy diagnoses according to type and anatomical site

Patient	Group	Type	Site
1	Gonadal Gastrointestinal	Squamous cells carcinoma Squamous cells carcinoma	Vulvae and cervix Anal/Rectal
2	Hematological	Hodgkin Lymphoma	Lymph node
3	Hematological	Non-Hodgkin lymphoma	Lymph node
4	Hematological	Non-Hodgkin lymphoma (MALT)	Orbital glands
5	Gonadal	Germ cell line teratoma	Testicle
6	Central Nervous System	Oligodendroglioma	Brain
7	Gastrointestinal	Adenocarcinoma	Sigmoid colon
8	Hematological	Acute lymphoblastic leukemia	Bone Marrow
9	Gonadal	Squamous cells carcinoma	Cervix
10	Gastrointestinal	Squamous cells carcinoma	Tongue
11	Gonadal	Squamous cells carcinoma	Vulvae
12	Gastrointestinal	Carcinoid	Stomach

By the time the diagnoses were confirmed, the clinical and laboratorial manifestations, that could be related to lupus activity, were: weight lost (5), hepatomegaly (2), adenomegaly (3), malar rash (3) and photosensitivity (2), arthritis (4), pleuritis (2), urine leucocytes (5) urine red cells (4), proteinuria (3) nephrotic proteinuria (2); only one of the cases had renal biopsy resulting in class V/III-S, with renal activity score 5/24 and chronicity 2/12, tubular infiltrates and atrophy in addition to granular immunofluorescent deposits of C1q, IgA, IgM and C3 in capillary loops.

Of the neuropsychiatric manifestations, one case had simultaneous demyelinating syndrome, psychosis and seizures, (1) headaches, (1) myelopathy, (1) mononeuritis, (1) unspecified visual compromising. Of the observed laboratorial parameters, lymphopenia (5), leukopenia (4), autoimmune haemolytic anaemia (4), thrombocytopenia (3), high ESR (9) with median values 43.5 (6–120) mm/h; high CRP, 1.57 (0.06–14) mg/L; low complement (4) with C3 median values 72.6 (0.74–135) mg/dL, C4 median values 13.6 (0.074–31.9) mg/dL. Of the specified positive ANA, homogeneous pattern (2), fine speckled (2), unspecified speckled (2) and dense granular (1); anti-DNA (8), anti-Smith (3), anti-RNP (3); lupus anticoagulant (2); anti-Ro (1); IgM anticardiolipin (1) were recorded in the series.

The Median (min–max) SLEDAI-2 K of all scores were 9 (0–38), of those 11 scored activity with median 10 (1–38). Eight patients presented a SLEDAI score > 4 and were considered in active disease status, 12.5 (7–38). Overall, it was scored in neuropsychiatric domain by seizures, psychosis and headaches; arthritis for musculoskeletal domain; malar rash, vasculitis and alopecia for skin domain; urine leukocytes, red blood cells, proteinuria and urine casts for renal; serositis with pleuritis and pericarditis; leukopenia and thrombocytopenia; high titres anti-dsDNA and low complement. Damage accrual scores median (min–max) scores for SLICC/ACR-DI (SDI) were 1 (1–5). Specific scores for damage domains were cataracts, peripheral neuropathy, transverse myelitis and persistent proteinuria. (Table 2).

**Table 2:** Description of SLICC/ACR-DI* scored at malignancy diagnosis in c-SLE cases

Domain	Descriptive	Frequency n (%)
Ocular	Cataract	1(8.3)
Neuropsychiatric	Peripheral neuropathy Myelitis	1(8.3) 1(8.3)
Renal	Persistent proteinuria > 3.5 g/day for more than 6 months	1(8.3)
Pulmonary	–	0(0)
Cardiovascular	Pericardiocentesis	1(8.3)
Peripheral vascular	–	0(0)
Gastrointestinal	–	0(0)
Musculoskeletal	–	0(0)
Skin	–	0(0)
Premature gonadal failure	–	0(0)
Diabetes	–	0(0)
Malignancy**	–	11(100)

^*^Systemic Lupus International Collaborating Clinics (SLICC)/American College of Rheumatology (ACR) Damage Index (SDI)

^**^One case was not scored due to less than 6 month follow up

Medication used during the whole disease course, with doses recorded by the time of malignancy diagnoses were: prednisone (8), median daily dose 12.5 (5–60) mg; hydroxychloroquine (10) median 4.1 (3–6.6) mg/kg/day. Only 5 used Mofetil Mycophenolate 19.2 (10–80.8) mg/kg/day. There was only one patient treated with each of the following: intravenous methylprednisolone (pulse), Azathioprine, intravenous Cyclophosphamide and Rituximab (Table 3).

**Table 3:** cSLE treatment received by the time of malignancy diagnoses

Drug	Fequency n of cases (%)	Doses Median
Prednisone or equivalent	8	12.5 mg/day
Intravenous methylprednisolone (Pulse)	1	1 g/day, 3 days
Hydroxychloroquine	10	4.1 mg/kg/day
Azathioprine	1	2.12 mg/kg/day
Mofetil mycophenolate	5	19.2 mg/kg/day
Intravenous cyclophosphamide	1	Monthly 0.5 g/m^2^
Rituximab	1	2 g/6 months

Malignancy diagnoses followed by transfer of care to Oncology was informed by the assistant physician in 11 cases. The reported patients received: chemotherapy (5), curative excision surgery (4), palliative care (1) or no treatment (1). Individual case description summary is presented on Table 4.

**Table 4:** Clinical and demographic descriptors for each of the c-SLE malignancy cases

Demographic and clinical characteristics	c-SLE Cases												
	1	2	3	4	5	6	7	8	9	10	11	12	Median (min–max)
Age at malignancy diagnosis (years)	24	14	12	16	15	13	32	30	27	32	27	22	23 (12–32)
Age at cSLE diagnosis	10	14	11	10	11	12	13	17	12	16	12	13	12 (10–17)
cSLE duration (years)	14	0.2	1.8	6.1	4	1.3	19	13	16	16	12	8.7	10 (0.2–19)
Gender (F:M)	F	F	F	F	M	F	F	F	F	F	F	F	11:1
Specific Malignancy	SC	HL	NHL	NHL	TT	OL	AC	ALL	SC	SC	SC	C	–
SLEDAI 2K scores	22	31	13	10	4	38	8	1	3	0	12	7	9 (0–38)
SLICC/ACR-DI scores	5	–	1	1	1	1	1	1	1	1	2	2	1 (1–5)
Drugs ever used during c-SLE follow up	PDN MPDN HXC MMF AZA CYC RTX	PDN MPDN HXC MMF AZA	PDN HXC AZA	PDN HXC AZA	PDN MPDN HXC AZA MMF CYC IVIG	PDN HXC	PDN HXC UNK	HXC UNK	PDN UNK	HXC MMF UNK	PDN HXC MMF UNK	PDN HXC MTX	

ALL, Acute lymphoblastic leukemia; AC, Adenocarcinoma; AZA, Azathioprine; c-SLE, childhood onset systemic lupus erythematosus; C, Carcinoid; CYC, Cyclophosphamide; HK, Hodgkin lymphoma; HXC, Hydroxychloroquine; IVIG, Intravenous Immunoglobulin; MTX, Methotrexate; MPDN, Methylprednisolone pulse; MMF, Mofetil mycophenolate; NHL, Non Hodgkin lymphoma; OL, Oligodendroglioma; PDN, Prednisone; RTX, Rituximab; SC, Squamous carcinoma; SLEDAI-2K, Systemic Lupus Erythematosus Disease Activity Index 2000; SLICC/ACR-DI, Systemic Lupus International Collaborating Clinics (SLICC)/American College of Rheumatology (ACR) Damage Index (SDI); TT, Gonadal Teratoma; UNK, Unknown data

## Discussion

We have identified malignancy frequency of 0.7% in a large retrospective series of cSLE, including, lymphohematopoietic and solid malignancy. Reports of metanalyses, systematic and scoping reviews or observational studies describing malignancy in cSLE [9, 14–16, 21, 22] were extensively reviewed. We highlight the importance of this work by describing rare events in cSLE, since pediatric malignancies represent 2 to 3% of malignancy rates in Brazil [23] and there were no previous reports in the same population.

Overall, the reported incidence of lymphomas in our population is 14%, in children and adolescents from 0 to 19 years (0.002%). [23]. There is higher incidence of non-Hodgkin lymphoma, regardless of age, in different reports estimated by Standard Incidence Ratio (SIR): 5.2 (CI 95% = 1.1–15.2) [8]; 18.6 (CI 95% = 3.84–54.4) [9] and 4.93 (CI 95% = 3.81–6.36) [14]. For Hodgkin lymphoma, 2.6 (CI 95% = 2.14–3.17) were reported [14]. Leukaemia makes 26% of all pediatric malignancy rates, from 0 to 19 years, with mean incidence of 0.004% [23]. There is a higher reported SIR of 2.01 (CI 95% = 1.64–2.47) in Leukaemia of adult lupus patients [14]. However, all the other comparisons, either of lymphohematopoietic malignancy or solid tumors, are very limited in the pediatric age. It was not possible to calculate the SIR in our series due to lack of data in the reference population of all the malignancies reported in our series.

In the Brazilian Malignancy Registry, carcinoma was the most frequently reported solid malignancy from 15 to 29 years, with the proportion of 14% over all malignancies [23]. In this category there were cervix, vulva, gastrointestinal and pharyngeal carcinomas. For the cervical carcinoma, the incidence rates were 0.0018% from 0 to 19 years of age [23]. SLE patients are susceptible to cervical carcinomas, estimated by SIR 1.56; 95% CI = 1.29–1.88 [14]; vulva, 3.48; 95% CI = 2.69–4.50 [14]; stomach, 1.31; 95% CI = 1.04–1.63 [14] and pharyngeal, 1.52; 95% CI = 1.0–2.3 [14]; based on reports of adults. Some of papillomavirus (HPV) associated malignancies are reported more often in SLE patients, although there are controversies about the associated risk factors. HPV routine immunization programme in our country only started in 2014, it was covered by the national immunization programme. Therefore, our cases were probably unvaccinated patients [24], reflecting natural disease course.

About CNS malignancy, we identified just one case (0.057%) of oligodendroglioma. Overall, CNS malignancy makes a group with common diagnosis in the paediatric age with incidence rates from 0 to 19 years of 0.002% [23]. However, there is no remarkable association of brain malignancy and SLE or c-SLE, SIR = 1.08; 95% CI = 0.64–1.81 [14], and one case was reported in large c-SLE malignancy association registry [22].

We found one case of gonadal malignancy (0,057%), a germ line cell testicle teratoma. Overall, germinative cells malignancy makes 3% in our reference population from 0 to 19 years, and 5% from 0 to 14 years. There is a trend of higher incidence in females [23], In our extensive search for meta-analyses, systematic reviews descriptive and observational studies [9, 14, 15, 21, 22] we did not identify SIR description for gonadal glands malignancy, in boys.

SLEDAI-2K scores were higher than those reported in adults with c-SLE and malignancy association, where the mean SLEDAI-2K score of 2, was observed.[17–20, 25]. In our previous analysis of 670 cases of c-SLE, in a sample from the same population, early damage accrual (SLICC/ACR-DI ≥ 1) occurred in 30%, comprising the renal, neuropsychiatric and muscle-skeletal domains [26]. In our series of c-SLE associated malignancy, in addition to the malignancy itself, scoring one point for each malignancy site, the other scored domains were ocular, neuropsychiatric, renal and cardiovascular domains. It might be possibly related to long disease duration of c-SLE cases diagnosed with malignancy [19].

Paraneoplastic syndrome is attributed when rheumatic symptoms appear up to two years before the malignancy diagnosis, although a precise definition is not established [28]. Three of our cases had an interval of less than two years from cSLE diagnoses and malignancy the and one of those had less than 6 month; and for that reason, damage score by SLICC/ACR-DI was not calculated.

Our report has limitations, first for the retrospective design, number of affected cases and overall population asymmetry, missing data, variable follow up duration, and a short window of observation, with limited control cases for data comparison. Ideally, prospective malignancy registry would provide more robust data for the c-SLE association, risk factors and outcome, but this is limited in our paediatric population.

Most of cases were young adults, by the time of malignancy diagnoses, either in the pediatric or adult clinic, depending on each of the services routine care [27]. Assistant physician informed the transfer of care to Oncology to start the malignancy care, and per protocol the rheumatology care was discontinued at the last follow up visit. Reports about treatment were provided by the Oncology care team.

Our series represent samples from all the regions population of the country, with universal access to care and a standardised practice, established in the registry. Although, it was not possible to compare the SIR from the reference population, due to lack of data in specific childhood malignancy registries, in particular for solid malignancy.

## Conclusion

The overall frequency of reported c-SLE malignancy was 0.7%. We observed intestinal, gonadal and cerebral malignancy rates and the high disease activity parameters observed by the time of cSLE-malignancy diagnoses. Malignancy suspicious awareness may have impact in clinical care.

## Data Availability

Data analyzed and biopsy reports, can be requested to the corresponding author, if necessary.

## Abbreviations

SLE: Systemic lupus erythematosus
cSLE: Childhood onset systemic lupus erythematosus
SLEDAI-2K: Systemic Lupus Erythematosus Disease Activity Index 2000
SLICC/ACR-DI (SDI): Systemic Lupus International Collaborating Clinics American College of Rheumatology Damage Index
CNS: Central nervous system
ACR: American College of Rheumatology
SIR: Standard Incidence Ratio
ESR: Erythrocyte sedimentation rate
CPR: C-reactive protein
HPV: Human papillomavirus

## References

[1] Hiraki LT, Benseler SM, Tyrrell PN, Hebert D, Harvey E, Silverman ED. Clinical and laboratory characteristics and long-term outcome of pediatric systemic lupus erythematosus: a longitudinal study. J Pediatr. 2008;152(4):550–6. 18346514 10.1016/j.jpeds.2007.09.019

[2] Silva CA, Avcin T, Brunner HI. Taxonomy for systemic lupus erythematosus with onset before adulthood. Arthritis Care Res. 2012;64(12):1787–93. 10.1002/acr.21757 PMC3463746 22730317

[3] Grivennikov SI, Greten FR, Karin M. Immunity, inflammation, and cancer. Cell. 2010;140(6):883–99. 10.1016/j.cell.2010.01.025. 20303878 10.1016/j.cell.2010.01.025 PMC2866629

[4] Noble PW, Bernatsky S, Clarke AE, Isenberg DA, Ramsey-Goldman R, Hansen JE. DNA-damaging autoantibodies and cancer: the lupus butterfly theory. Nat Rev Rheumatol. 2016;12(7):429–34. 10.1038/nrrheum.2016.23. 27009542 10.1038/nrrheum.2016.23

[5] Bernatsky S, Joseph L, Boivin JF, Gordon C, Urowitz M, Gladman D, et al. The relationship between cancer and medication exposures in systemic lupus erythaematosus: a case-cohort study. Ann Rheum Dis. 2008;67(1):74–9. 17545189 10.1136/ard.2006.069039

[6] Gladman DD, Goldsmith CH, Urowitz MB, Bacon P, Fortin P, Ginzler E, et al. The systemic lupus international collaborating clinics/American college of rheumatology (SLICC/ACR) damage index for systemic lupus erythematosus international comparison. J Rheumatol. 2000;27(2):373–6. 10685799

[7] Gladman D, Ginzler E, Goldsmith C, Fortin P, Liang M, Urowitz M, Bacon P, Bombardieri S, Hanly J, Hay E, Isenberg D, Jones J, Kalunian K, Maddison P, Nived O, Petri M, Richter M, Sanchez-Guerrero J, Snaith M, Sturfelt G, Symmons D, Zoma A. The development and initial validation of the systemic lupus international collaborating clinics/American College of Rheumatology damage index for systemic lupus erythematosus. Arthritis Rheum. 1996;39(3):363–9. 10.1002/art.1780390303. 8607884 10.1002/art.1780390303

[8] Bernatsky S, Ramsey-Goldman R, Labrecque J, Joseph L, Boivin JF, Petri M, et al. Cancer risk in systemic lupus: an updated international multi-centre cohort study. J Autoimmun. 2013;42:130–5. 10.1016/j.jaut.2012.12.009. 23410586 10.1016/j.jaut.2012.12.009 PMC3646904

[9] Bernatsky S, Clarke AE, Niaki OZ, Labrecque J, Schanberg LE, Silverman ED, et al. Malignancy in pediatric-onset systemic lupus erythematosus. J Rheumatol. 2017;44(10):1484–6. 28765255 10.3899/jrheum.170179 PMC5657309

[10] Goobie GC, Bernatsky S, Ramsey-Goldman R, Clarke AE. Malignancies in systemic lupus erythematosus: a 2015 update. Curr Opin Rheumatol. 2015;27(5):454–60. 26125105 10.1097/BOR.0000000000000202 PMC4562287

[11] Lu M, Bernatsky S, Ramsey-Goldman R, Petri M, Manzi S, Urowitz MB, et al. Non-lymphoma hematological malignancies in systemic lupus erythematosus. Oncol. 2013;85(4):235–40. 10.1159/000350165 PMC3880772 24107608

[12] Ni J, Qiu LJ, Hu LF, Cen H, Zhang M, Wen PF, et al. Lung, liver, prostate, bladder malignancies risk in systemic lupus erythematosus: evidence from a meta-analysis. Lupus. 2014;23(3):284–92. 24429300 10.1177/0961203313520060

[13] Chang SH, Park JK, Lee YJ, Yang JA, Lee EY, Song YW, et al. Comparison of cancer incidence among patients with rheumatic disease: a retrospective cohort study. Arthritis Res Ther. 2014;16(4):1–6. 10.1186/s13075-014-0428-x PMC4295295 25163486

[14] Song L, Wang Y, Zhang J, Song N, Xu X, Lu Y. The risks of cancer development in systemic lupus erythematosus (SLE) patients: a systematic review and meta-analysis. Arthritis Res Ther. 2018;20(1):270. 10.1186/s13075-018-1760-3. 30522515 10.1186/s13075-018-1760-3 PMC6282326

[15] Cao L, Tong H, Xu G, Liu P, Meng H, Wang J, et al. Systemic lupus erythematous and malignancy risk: a meta-analysis. PLoS ONE. 2015;10(4):1–21. 10.1371/journal.pone.0122964 PMC4401738 25885411

[16] Da Silva Aoki PR, El Dib R, Silva CAA, Magalhaes CS. Description of malignancy rates in childhood- and adult-onset systemic lupus erythematous by proportional meta-analysis. J Clin Rheumatol. 2017;23(4):187–92. 28492421 10.1097/RHU.0000000000000551

[17] Fiorot FJ, Islabão AG, Pereira RM, Terreri MT, Saad-Magalhães C, Novak GV, et al. Disease presentation of 1312 childhood-onset systemic lupus erythematosus: influence of ethnicity. Clin Rheumatol. 2019;38(10):2857–63. 31209708 10.1007/s10067-019-04631-0

[18] Novak GV, Molinari BC, Ferreira JC, Sakamoto AP, Terreri MT, Pereira RMR, et al. Characteristics of 1555 childhood-onset lupus in three groups based on distinct time intervals to disease diagnosis: a Brazilian multicenter study. Lupus. 2018;27(10):1712–7. 30020023 10.1177/0961203318787037

[19] Lopes SRM, Gormezano NWS, Gomes RC, Aikawa NE, Pereira RMR, Terreri MT, et al. Outcomes of 847 childhood-onset systemic lupus erythematosus patients in three age groups. Lupus. 2017;26(9):996–1001. 28134038 10.1177/0961203317690616

[20] Gomes RC, Silva MF, Kozu K, Bonfá E, Pereira RM, Terreri MT, et al. Features of 847 childhood-onset systemic lupus erythematosus patients in three age groups at diagnosis: a Brazilian multicenter study. Arthritis Care Res. 2016;68(11):1736–41. 10.1002/acr.22881 27014968

[21] Huang HB, Jiang SC, Han J, Cheng QS, Bin DC, Pan CM. A systematic review of the epidemiological literature on the risk of urological cancers in systemic lupus erythematosus. J Cancer Res Clin Oncol. 2014;140(7):1067–73. 24525705 10.1007/s00432-014-1604-8 PMC11824115

[22] Bernatsky S, Clarke AE, Labrecque J, von Scheven E, Schanberg LE, Silverman ED, et al. Cancer risk in childhood-onset systemic lupus. Arthritis Res Ther. 2013;15(6):2–5. 10.1186/ar4388 PMC3978586 24267155

[23] Brasil. Ministério da Saúde.Instituto Nacional do Câncer (INCA). Incidência , mortalidade e morbidade hospitalar por câncer em crianças , adolescentes e adultos jovens no Brasil. 2016. https://bvsms.saude.gov.br/bvs/publicacoes/incidencia_mortalidade_hospitalar_cancer_criancas_adolescentes_adultos_jovens_brasil.pdf. Accessed 15 Aug 2023

[24] Rotstein Grein IH, Pinto NF, Lobo A, Groot N, Sztajnbok F, da Silva CAA, et al. Safety and immunogenicity of the quadrivalent human papillomavirus vaccine in patients with childhood systemic lupus erythematosus: a real-world interventional multi-centre study. Lupus. 2020;29(8):934–42. 32501172 10.1177/0961203320928406

[25] Guo J, Ren Z, Li J, Li T, Liu S, Yu Z. The relationship between cancer and medication exposure in patients with systemic lupus erythematosus: a nested case-control study. Arthritis Res Ther. 2020;22(1):159. 32586407 10.1186/s13075-020-02228-6 PMC7318532

[26] Pitta AC, Silva CA, Insfrán CE, Pasoto SG, Trindade VC, Novak GV, et al. The new 2019-EULAR/ACR classification criteria specific domains at diagnosis can predict damage accrual in 670 childhood-onset systemic lupus erythematosus patients. Lupus. 2021;30(14):2286–91. 34689652 10.1177/09612033211054397

[27] Silva CA, Terreri MT, Bonfa E, Saad-Magalhães C. Pediatric rheumatic disease patients: time to extend the age limit of adolescence? Adv Rheumatol. 2018;58(1):30. 30657082 10.1186/s42358-018-0031-y

[28] Manger B, Schett G. Paraneoplastic syndromes in rheumatology. Nat Rev Rheumatol. 2014;10(11):662–70. 10.1038/nrrheum.2014.138. 25136782 10.1038/nrrheum.2014.138

